# Dietary energy alters jejunal microbial function without changing its structure in small-tailed Han sheep

**DOI:** 10.3389/fvets.2026.1730873

**Published:** 2026-03-05

**Authors:** Yize Song, Xiufen Pu, Qing Liu, Senxuan Hou, Dongbin Zou, Yuping Xiang, Shiyu Gu, Mingxing Chu

**Affiliations:** State Key Laboratory of Animal Biotech Breeding, Institute of Animal Science, Chinese Academy of Agricultural Sciences, Beijing, China

**Keywords:** different dietary energy levels, functional potential, jejunal microbiota, metagenomic sequencing, sheep

## Abstract

Dietary energy levels typically influence the structure and functional profile of the gastrointestinal microbial community. In this study, thirty 6-month-old Small-tailed Han (STH) sheep were randomly divided into three groups and fed corn-based diets with different energy levels for 150 days. Jejunal contents were then collected and analyzed using metagenomic sequencing to assess microbial alpha diversity and taxonomic composition. Functional annotation and enrichment analysis were performed using the KEGG database. Principal coordinate analysis (PCoA) and alpha diversity indices (Chao1, Shannon, Simpson and good coverage) revealed no significant changes in the overall structure or macro-ecological characteristics of the jejunal microbial community in response to dietary energy levels. At the phylum level, Bacillota was the absolutely dominant phylum, while at the genus level, Methanobrevibacter was the most abundant genus. The abundances of these core microbial taxa did not differ significantly among groups. However, KEGG functional enrichment analysis revealed significant differences in microbial functions between groups. The low-energy group exhibited enrichment in pathways related to energy deficiency and stress adaptation, whereas the high-energy group showed significant enrichment in pathways associated with active growth and anabolic metabolism. In conclusion, although dietary energy levels did not significantly alter the microbial community structure in the jejunum of STH sheep, they profoundly influenced its functional potential. These findings suggest that dietary energy may modulate host nutrient acquisition and health status by regulating the functional characteristics of the jejunal microbiota.

## Introduction

Obesity is defined as a metabolic syndrome caused by the abnormal accumulation of body fat and has emerged as a worldwide public health problem (1). Concerning human health, obesity induces dyslipidemia, thereby promoting the development of cardiovascular diseases, cancer, and diminished fertility in females (2–4). In the context of animal production, it leads to impaired reproductive performance, reduced lactation ability, and smaller litter sizes (5). Consequently, the regulation of bodily fat deposition and the reduction of obesity hold substantial importance for animal production.

Adipose tissue is instrumental in the body’s energy regulation. An excess of energy is converted into and stored as neutral triglycerides in adipose tissue, consequently contributing to the development of obesity (6). Thus, energy metabolism represents a significant factor in modulating obesity. Acting as a crucial interface between dietary intake and host physiology, the gut microbiota exhibits strong correlations with body fat content, energy metabolism, and immune function (7, 8). It has been documented that the abundance of Bifidobacterium is diminished in obese mouse models (9), and the transplantation of gut microbiota from obese human donors into germ-free mice induces an obese phenotype in the latter (10).

The intestine is the primary site for nutrient absorption, and its resident microbiota profoundly influences growth and development as well as production performance in adulthood (11). Studies have shown that dietary energy restriction reduces the relative abundance of beneficial bacteria (12). The intestinal microbial community in ruminants is positively correlated with dietary energy levels. Providing a high-energy diet to ewes has been shown to enhance intestinal villus height and crypt depth, concurrently upregulating the abundance of genes associated with carbohydrate metabolism (13). An increase in dietary energy density can improve the physical, chemical, and immune barrier functions of the ovine small intestine, an effect that is potentially implicated in restructuring of the gut microbiota (14). Conversely, several studies indicate that elevated dietary energy concentrations may induce gut microbial dysbiosis, compromise barrier integrity, and provoke inflammatory responses in both goats and sheep (15, 16).

Dietary energy concentration is a key factor influencing intestinal function and the gut microbiota. Most studies on the effects of energy levels in the digestive tract of ruminants have focused on ruminal or hindgut fermentation, with little reported on the small intestine. In summary, dietary energy level is a key factor regulating the interaction between gut microbiota and the host, yet its specific mechanisms of action on the jejunal microbiota in ruminants remain unclear. To address this, we used Small-tailed Han sheep as a model to systematically investigate the effects of different energy-level diets on the composition and function of the jejunal microbiota through metagenomic approaches.

## Materials and methods

### Sample collection

The 30 STH sheep (6 months old) used in this study were randomly divided into three groups, with 10 sheep in each group. According to the grouping, the sheep in each group were fed corn-based diets with different proportions to create differences in digestible energy intake among the groups (Supplementary Table S1). Throughout the trial, all sheep were maintained under the same feeding and management conditions and were allowed free access to water. At the end of the experiment, all sheep were slaughtered for carcass weight measurement (Supplementary Table S2). Jejunal content samples were collected from the intestinal segment approximately 1 to 1.5 meters distal to the pylorus. DNA was extracted from the collected jejunal content for metagenomic sequencing.

### Data quality control and statistics

After the sequencing results were generated, raw data were processed using fastp for adapter removal and elimination of low-quality reads. Metrics such as raw sequencing volume, valid sequencing volume, Q30, and GC content were calculated for comprehensive quality assessment. Potential host-derived reads were filtered out using bbmap software.

### Sequence assembly

After quality control, the optimized sequences were assembled using MEGAHIT, a software based on the De Bruijn graph principle. By leveraging overlaps between k-mers, a De Bruijn graph was constructed to generate contigs. Contigs longer than 500 bp were selected for statistical analysis and subsequent studies.

### Data analysis

Alpha diversity analysis was performed by calculating alpha diversity indices based on species-level read counts, followed by assessing the significance of differences in these indices across groups. Inter-group differences in alpha diversity indices were assessed using the Kruskal-Wallis test, and when a significant difference was observed (*p* < 0.05), Dunn’s post-hoc test was applied for pairwise comparisons.

Using Salmon software, clean reads from each sample were aligned to a non-redundant gene set to quantify gene abundance in the corresponding samples. Genes with fewer than 2 reads across all samples were removed, resulting in a final set of Unigenes. Based on the number of mapped reads and gene length, abundance information for each gene in each sample was calculated.

To examine whether species exhibited significant differences between groups, hypothesis testing was performed on species abundance data at different taxonomic levels using the Wilcoxon rank-sum test (for two groups) or the Kruskal-Wallis test (for three or more groups). For comparisons involving three or more groups, Dunn’s post-hoc test was subsequently conducted when the overall *p*-value was < 0.05 to identify which specific group pairs differed significantly. A *p*-value < 0.05 was considered statistically significant. Species showing significant differences were selected, and the top 10 most abundant ones were visualized using boxplots.

Functional annotation was carried out by aligning non-redundant genes against various functional databases using DIAMOND, retaining hits with e-values < 1e-5 and selecting proteins with the highest sequence similarity. For each sequence, the alignment result with the highest score (one HSP > 60 bits) was chosen for downstream analysis. Relative abundances were summarized at different functional levels, with KEGG pathways categorized into six hierarchies.

To identify significantly different functional biomarkers between groups, rank-sum tests (Kruskal-Wallis test followed by Dunn’s post-hoc test for multi-group comparisons) were applied to detect differential functions, followed by Linear Discriminant Analysis (LDA) for dimensionality reduction and evaluation of effect sizes, yielding LDA scores.

## Results

### Jejunal microbiota diversity analysis

**Figure 1:**
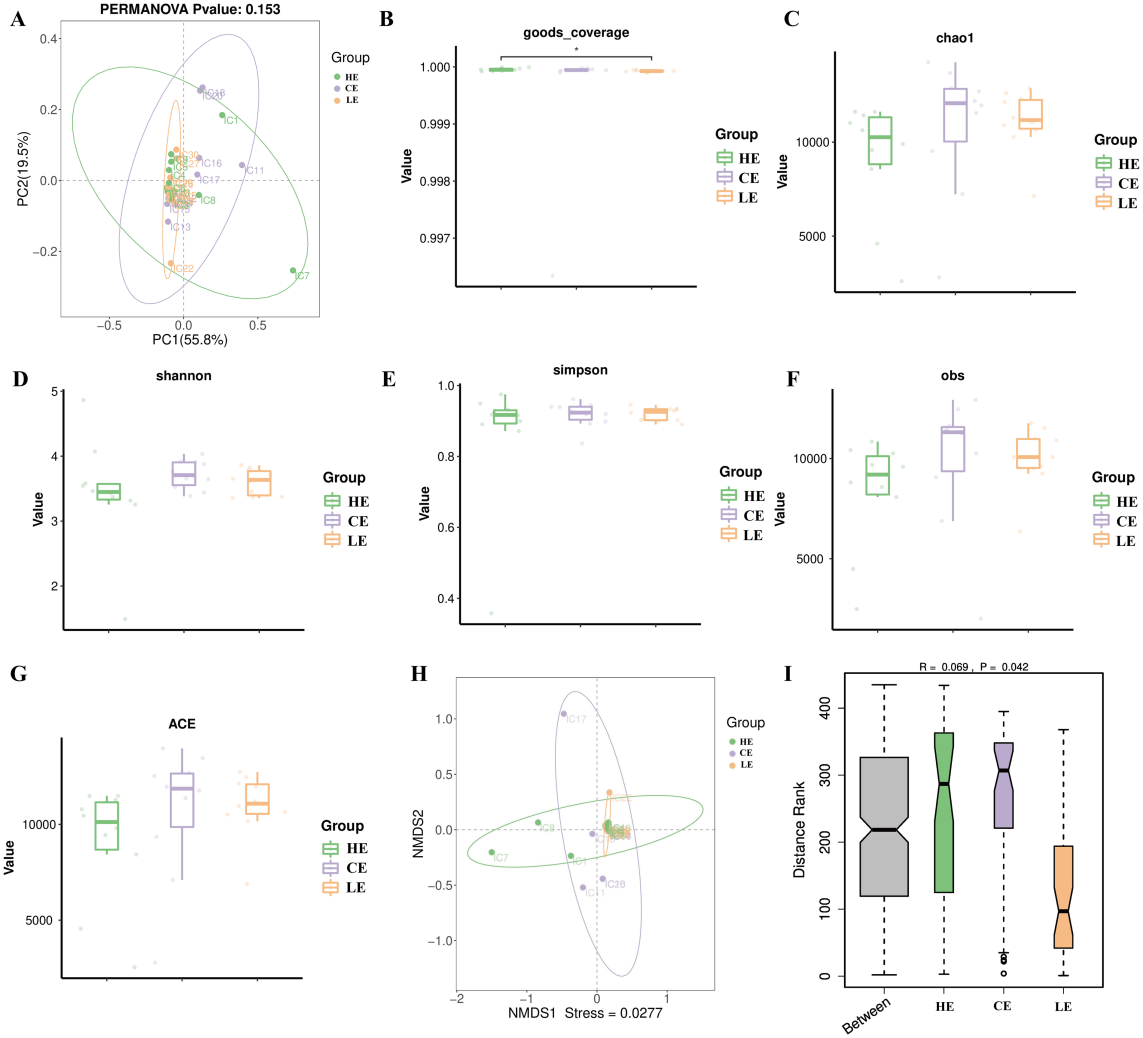
Analysis of jejunal microbiota diversity in STH sheep. **(A)** PCoA analysis at the genus level; **(B–G)** *α*-diversity of the jejunal microbiota (goods coverage, Chao1, Simpson, Obs, and Ace indices); **(H,I)** *β*-diversity of the jejunal microbiota (NMDS and Anosim analysis).

To assess the impact of different levels of energy feed on the overall structure of the jejunal microbial community in STH sheep, we conducted Principal Coordinate Analysis (PCoA) at genus (Figure 1A) levels. The results showed that, based on the current data and analytical methods, there was no significant spatial separation trend between sample points from the different groups. To further investigate the effect of energy level on the microbiota, we calculated alpha diversity indices based on species-level read counts to evaluate the species diversity and richness within the jejunal microbial communities of the different groups. The results indicated that the Goods coverage values for all samples exceeded 0.999 (Figure 1B), demonstrating that the current sequencing depth was sufficient to capture the vast majority of microbial species in the samples. However, the different levels of energy feed did not cause significant changes in the macro-ecological characteristics of the jejunal microbial community, including community richness (Chao1) (Figure 1C) and diversity (Shannon/Simpson) (Figures 1D,E). There was no statistically significant difference in Obs and ACE among the three groups (Figures 1F,G). Beta diversity analysis illustrated the composition of the microbial communities among the three groups. However, NMDS and ANOSIM analyses did not reveal significant separation between them (Figures 1H,I). This suggests that the influence of this factor on the jejunal microbial community is not a broad ecological restructuring, but may manifest as more subtle and specific changes.

### Jejunal microbial composition

**Figure 2:**
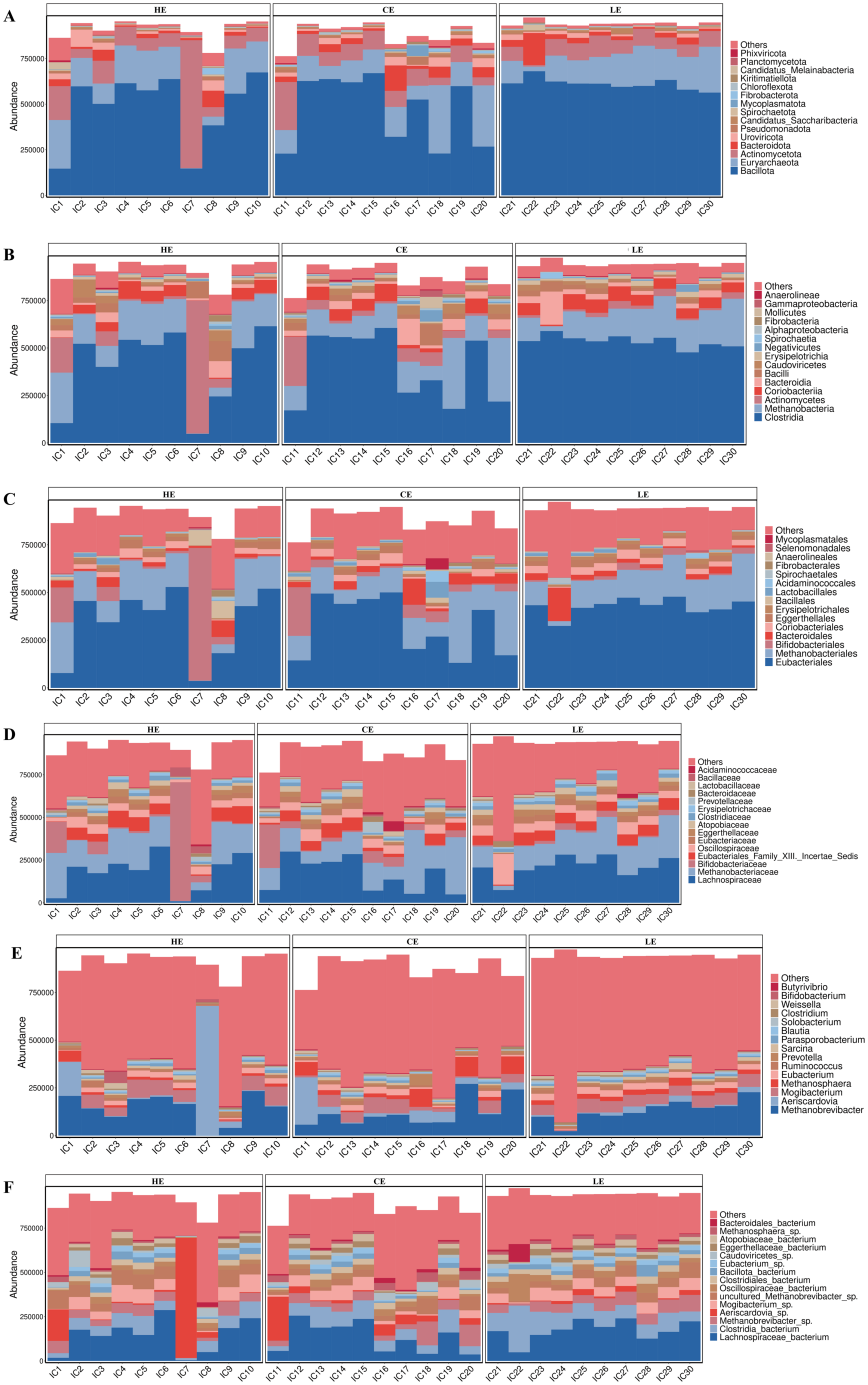
Microbial diversity analysis in the jejunum **(A–F)**. Entries labeled as _bacterium represent sequences that could not be matched to definitive species names, typically indicating uncultured or genomically poorly characterized taxa.

Species annotation was obtained using the taxonomic database corresponding to the NR database. The abundance of each species was calculated using the sum of the gene abundances corresponding to that species. A total of 204 phyla, 220 classes, 452 orders, 1,045 families, 4,027 genera, and 22,272 species were annotated (Supplementary Tables S3,S4). At the phylum level, Bacillota was the most abundant phylum with a mean relative abundance of 56.7%, followed by Euryarchaeota (17.7%), Actinomycetota (12.2%), and Bacteroidota (4.2%) (Figure 2A). Together, these four phyla accounted for approximately 90% of the total microbial community. At the class level, the most abundant microorganisms were Methanobacteria and Clostridia (Figure 2B). At the order level, they were Eubacteriales and Methanobacteriales (Figure 2C). Lachnospiraceae and Methanobacteriaceae were the dominant families at the family level (Figure 2D). At the genus level, Methanobrevibacter exhibited absolute predominance within the microbial community, with Aeriscardovia and Mogibacterium being the subsequent most abundant genera (Figure 2E). At the species level, Lachnospiraceae_bacterium, Clostridia_bacterium, and Methanobrevibacter_sp were the three most abundant microorganisms (Figure 2F). At the species level, many taxonomic units could only be annotated to higher taxonomic ranks (e.g., family or genus), reflecting both the limitations of current databases and the presence of a substantial number of uncultured microorganisms in the environment.

### Differential analysis of jejunal microbiota

**Figure 3:**
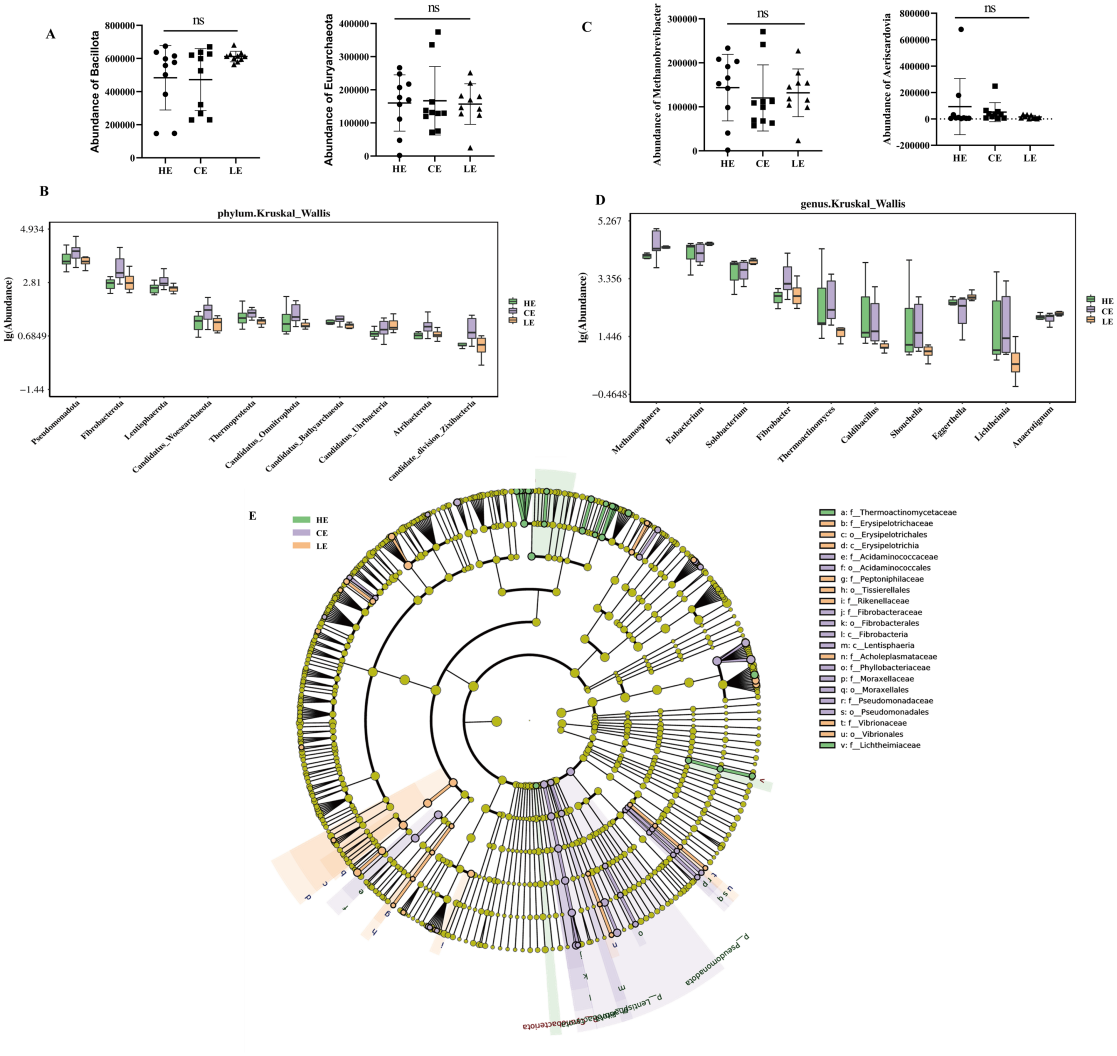
Differential analysis of jejunal microbiota. **(A)** Abundances of *Bacillota* and *Euryarchaeota* showed no significant differences among groups; **(B)** boxplot of the top 10 most abundant significantly different species; **(C)** abundances of *Methanobrevibacter* and *Aeriscardovia* showed no significant differences among groups; **(D)** boxplot of the top 10 most abundant significantly different species; **(E)** LEfSe analyzed the relative abundance of differential species among the three communities. Linear discriminant analysis (LDA) effect size (LEfSe) was used to identify microbial taxa with statistically significant differences among groups (LDA score > 2.0). Only taxonomic units with an LDA score > 2.0 are displayed in the figure.

We compared the abundances of Bacillota and Euryarchaeota across the different groups at the phylum level. As the energy level in the feed changed, no statistically significant differences were observed in the abundances of Bacillota and Euryarchaeota (Figure 3A). However, to identify species with significant differences among the groups, hypothesis testing was performed on inter-group species abundance data using the Kruskal-Wallis method. Species showing significant differences were screened based on *p*-values, and the top 10 most abundant significant species were used to generate box plots. The results demonstrated that Pseudomonadota, Fibrobacterota, and Lentisphaerota were ranked as the top three phyla exhibiting significant differences across the groups (Figure 3B). When the abundances of these predominant genera, Methanobrevibacter and Aeriscardovia, were compared across groups, no statistically significant alterations were observed in response to graded dietary energy levels (Figure 3C). Box plots were constructed for the top 10 most abundant significant species, identifying Methanosphaera, Eubacterium, and Solobacterium as the three leading genera displaying significant differences across the experimental groups (Figure 3D). This indicates that the different levels of energy feed did not induce macroscopic or global alterations in the jejunal microbial community of STH sheep; instead, a highly stable core community structure was maintained. We employed LEfSe to analyze the differentially abundant taxa among the three groups. The results revealed that the family Thermoactinomycetaceae was most abundant in the HE group. The family Erysipelotrichaceae, order Erysipelotrichales, and class Erysipelotrichia were more enriched in the LE group compared to the other two groups. In contrast, the family Acidaminococcaceae and order Acidaminococcales exhibited the highest abundances in the CE group (Figure 3E).

### Function of the jejunal microbiome

**Figure 4:**
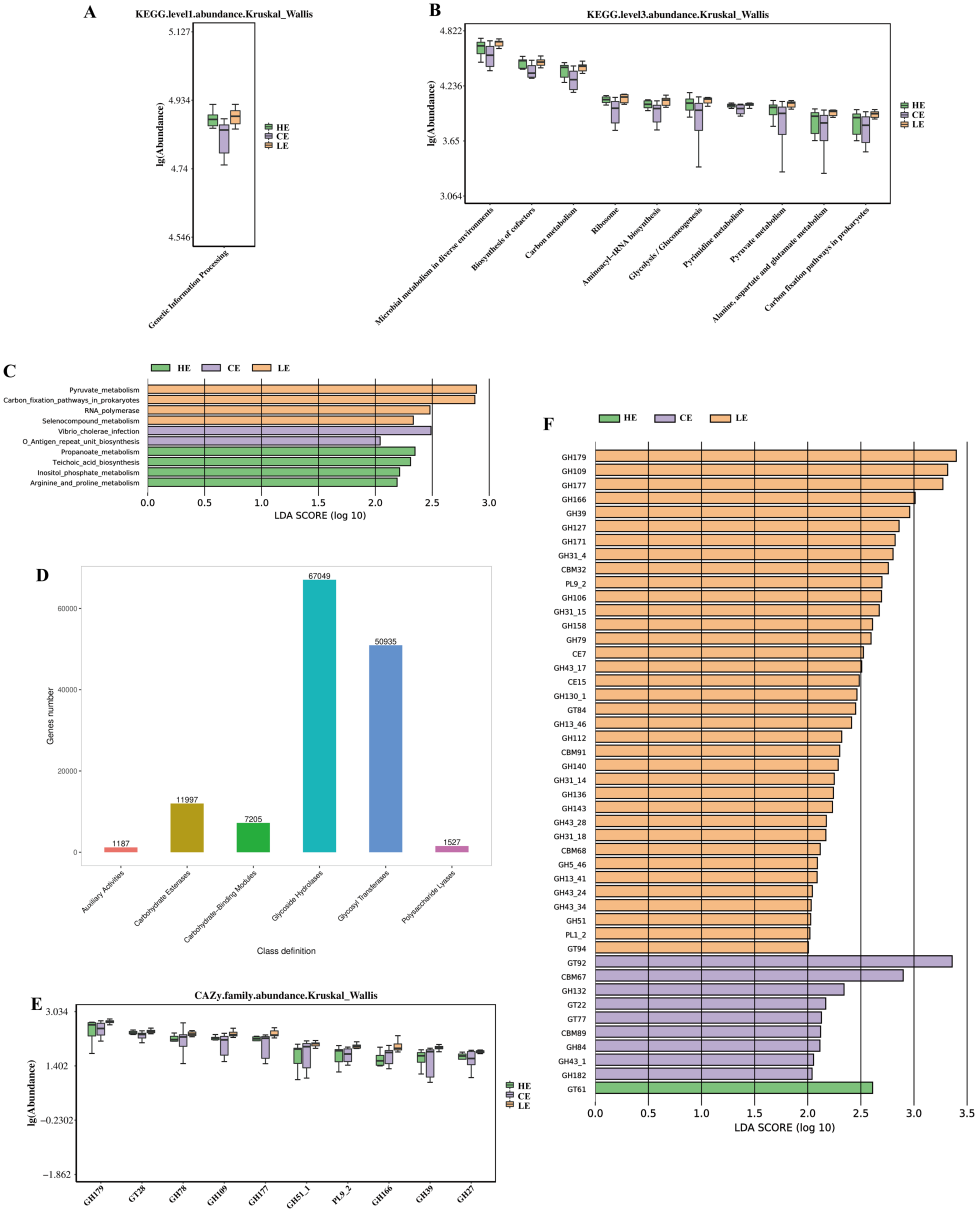
Functional level analysis of the jejunal microbiota. **(A)** KEGG enrichment results at level 1; **(B)** KEGG enrichment results at level 3; **(C)** Bar chart of LDA scores showing statistically different biomarkers between groups, where the bar length represents the effect size of the differential functions (i.e., LDA score); **(D)** annotation of the CAZy database across six major functional categories; **(E)** CAZy analysis of the top 10 most significantly differential carbohydrate-active enzymes; **(F)** Screening by LEfSe analysis for functional carbohydrate enzymes that were significantly different among groups.

Despite the robust macroscopic stability of the jejunal microbiota in response to varying dietary energy levels, we investigated whether significant functional differences existed among the groups based on functional abundance tables at different levels. KEGG pathway enrichment analysis revealed significant differences in “Genetic Information Processing” at Level 1 (Figure 4A). At Level 3, significant pathways included “Microbial metabolism in diverse environments”, “Biosynthesis of cofactors”, and “Carbon metabolism” (Figure 4B). To identify distinct functional biomarkers across the groups, we generated an LDA score plot, as shown in the distribution chart of LDA values for differential functions. In the low-energy group, the differential biomarkers were Pyruvate metabolism, Carbon fixation pathways in prokaryotes, RNA polymerase, and Selenocompound metabolism. The normal diet group was characterized by *Vibrio cholerae* infection and O Antigen repeat unit biosynthesis. In the high-energy group, the biomarkers identified were Propanoate metabolism, Teichoic acid biosynthesis, Inositol phosphate metabolism, and Arginine and proline metabolism (Figure 4C). Furthermore, we used the CAZy database to analyze the microbial contributions to carbohydrate metabolism. The annotation results were categorized, revealing that Glycoside Hydrolases (GHs) had the highest number of annotated genes, reaching 67,049 (Figure 4D). Additionally, we visualized the top 10 carbohydrate-active enzymes with the most significant differences between groups (Figure 4E). Moreover, when the LDA score was set above 2, glycoside hydrolases (GHs) were most abundant in the low-energy group, while only glycosyltransferase 61 (GT61) was annotated in the high-energy group (Figure 4F).

## Discussion

The intestine is the primary site for nutrient absorption, and its optimal function relies on its resident microbiota. Conversely, microbial colonization is itself regulated by intestinal function (17). The composition and diversity of the gut microbiota play a crucial role in maintaining intestinal balance and homeostasis (18). In this study, we first conducted PCoA at the phylum and genus levels. Our analysis did not detect statistically significant differences in the overall structure of the microbial communities among the different groups. Secondly, macroscopic metrics of the gut microbiota, including PCoA, Chao1, Shannon, and Simpson indices, showed no significant differences between groups. Dietary energy level had no significant impact on the evenness or richness of the gut microbial community, and the microbial structure did not differ significantly among treatments. These results indicate that varying levels of energy feed had a limited impact on the jejunal microbiota of STH sheep. This suggests that the influence of this factor does not constitute a broad ecological restructuring but rather maintains a highly stable core community structure. It may have only affected a few key bacteria, viruses, or fungi, or altered the functional potential of the microbial population without changing its taxonomic composition.

At the phylum level, the dominant microbial community in the jejunum of STH sheep in this study was Bacillota, which is consistent with findings from studies on the bovine gut by Durso et al. (19) and Marcelo et al. (20). The dominance of Bacillota remained absolute regardless of dietary energy variations. This stability suggests it constitutes a core characteristic of the microbial community at this site, likely crucial for maintaining basic jejunal function. At the genus level, the top three most abundant genera were Methanobrevibacter, Aeriscardovia, and Mogibacterium. In the ileum of newborn calves, Methanosarcina is one of the most abundant archaeal genera (21). In studies on Tibetan sheep, Methanobrevibacter has been identified as a key microbe involved in regulating meat color, muscle fiber characteristics, and amino and fatty acid content (22). However, in ruminants, the rumen serves as the primary habitat for methanogens. As digesta moves, microorganisms (including Methanosarcina attached to particles) are continuously transferred from the rumen downstream. Therefore, the high abundance of the Methanobrevibacter genus in the jejunum likely reflects continuous microbial input originating from the rumen, rather than a locally proliferating microbial community. Aeriscardovia plays an important role in preventing intestinal infections, lowering cholesterol levels, and stimulating the immune system (23). Interestingly, among these, only Mogibacterium belongs to Bacillota. This observation appears to corroborate the PCoA and alpha diversity results, indicating that dietary energy levels did not cause a broad ecological restructuring of the jejunal microbiota but likely only affected a few key microbial taxa.

As the primary site for nutrient absorption, the jejunum’s microbial community is positioned to influence the final stages of energy harvest from digesta (24). The investigation into functional biomarkers within the jejunal microbiota yielded compelling insights, demonstrating that microbial functions underwent pronounced and specific adaptations to dietary energy levels, despite the absence of macroscopic shifts in taxonomic composition. Under conditions of low dietary energy, the community exhibited a phenotype oriented toward survival and maintenance. Significant enrichment of Pyruvate metabolism, a cornerstone of central energy metabolism (25), reflects enhanced microbial effort to harness energy from scarce substrates for sustaining basal cellular functions under stringent energy deprivation. The enrichment of pathways related to pyruvate metabolism and carbon fixation in the low-energy group suggests a microbial strategy to maximize energy extraction from limited substrates (26), potentially aiding the host in maintaining energy homeostasis under restrictive conditions. Notably, the enrichment of Carbon fixation pathways in prokaryotes (27) points to a potential microbial capability to assimilate inorganic carbon (e.g., CO₂), representing an “open-source” metabolic tactic activated during nutrient stress. Collectively, the low-energy group’s microbiota was defined by an “energy-starved” and “nutrient-stressed” phenotype, prompting the activation of diverse core metabolic and alternative energy-harvesting pathways to mitigate the energy deficit. The stress adaptation pathways enriched in the low-energy group primarily respond to “nutrient starvation stress”. This directly results from the prolonged deficiency of available energy substrates in the diet.

Conversely, the core characteristics of the jejunal microbiota under high dietary energy levels were hallmarked by “robust growth” and “active synthesis,” whereby the microbes efficiently transformed the plentiful energy substrates into both microbial biomass and host-beneficial metabolic products. Propanoate represents a significant short-chain fatty acid that functions as an energy source and an important host signaling molecule (28), and teichoic acids are essential structural components of the Gram-positive bacterial cell wall (29). The high-energy group’s enrichment in propanoate metabolism and biosynthetic pathways could enhance the supply of microbially derived metabolites such as short-chain fatty acids (SCFAs) (30), which serve as important energy sources and signaling molecules for the host. The finding that Propanoate metabolism and Teichoic acid biosynthesis were concurrently enriched demonstrates that the microbiota exhibited accelerated growth and replication under high-energy conditions, concomitant with an increased output of host-advantageous metabolites. Pathways such as Inositol phosphate metabolism (31) and Arginine and proline metabolism (32, 33), implicated in signal transduction and stress resistance, were presumably requisite for preserving intracellular homeostasis within this nutrient-abundant milieu. In conclusion, dietary energy levels potentially modulate host nutritional harvest, health status, and productive performance through direct regulation of the functional profile of the jejunal microbiota.

The observed shift in microbial functional potential, particularly under different dietary energy levels, may have direct implications for host energy balance and overall productivity (24, 26, 30). While this study did not directly measure host metabolic outcomes, these functional adaptations suggest that dietary energy levels may modulate host energy availability and utilization through the jejunal microbiota, with potential consequences for growth efficiency, feed conversion, and overall production performance in ruminants. The functional changes observed in this study occurred against a backdrop of stable macro-scale community structure. This ‘structure–function decoupling’ suggests that the driving factors may reside at a more micro-scale level, such as functional gene regulation within core taxa or dynamic changes in rare species. Future studies employing higher-resolution approaches, such as metatranscriptomics or strain-resolved binning, could further elucidate the specific mechanisms involved.

## Conclusion

This study revealed a distinct pattern of how dietary energy levels influence the jejunal microbiota in STH sheep through metagenomic analysis. Although diets with different energy levels did not significantly alter the overall structure or alpha diversity of the jejunal microbial community, functional enrichment analysis demonstrated significant and specific adaptive changes in microbial metabolic potential. The low-energy diet enriched pathways related to energy stress and survival maintenance, such as pyruvate metabolism and carbon fixation, while the high-energy diet significantly enhanced functions associated with bacterial growth and biosynthesis, including propanoate metabolism, teichoic acid biosynthesis, and inositol phosphate metabolism. This study highlights that dietary energy levels significantly altered the functional potential of the jejunal microbiota in STH sheep without substantially changing its macro-taxonomic composition. Future nutritional strategies aimed at improving ruminant production performance through microbiota modulation should focus not merely on altering microbial taxa but rather on how to precisely regulate their metabolic functions.

## Data Availability

The data presented in the study are deposited in the China National Center for Bioinformation repository, accession number PRJCA048502 (https://ngdc.cncb.ac.cn/bioproject/browse/PRJCA048502).
