# Supplementary material for: Dietary energy alters jejunal microbial function without changing its structure in small-tailed Han sheep
Source: Front Vet Sci. 2026 Mar 5;13:1730873. doi: 10.3389/fvets.2026.1730873 (PMC13021366; doi:10.3389/fvets.2026.1730873)
Supplement: Supplementary file 1 [file Data_Sheet_1.zip › Supplementary Data Sheet 1/Table S1-S3.DOCX]

**Table S1. Measurable digestible energy of the diet in different groups of STH sheep.**

| Groups | Digestible energy (DE), MJ/kg |
| --- | --- |
| High-energy | 10.8 |
| Control-energy | 9.5 |
| Low-energy | 8.2 |

**Table S2. Carcass weight of STH sheep across different groups.**

| Groups | Carcass weight, kg |
| --- | --- |
| High-energy | 32.48±1.69 |
| Control-energy | 24.80±1.57 |
| Low-energy | 20.39±0.94 |

**Note: Carcass weight values in the table are presented as mean ± SEM.**

**Table S3. Statistical analysis of STH sheep jejunal microbiota at different taxonomic levels is presented.**

| Taxonomic Hierarchy | Numbers |
| --- | --- |
| Phylum | 204 |
| Class | 220 |
| Order | 452 |
| Family | 1045 |
| Genus | 4027 |
| Species | 22272 |
